# Supplementary material for: An In-Depth Characterization of SARS-CoV-2 Omicron Lineages and Clinical Presentation in Adult Population Distinguished by Immune Status
Source: Viruses. 2025 Apr 8;17(4):540. doi: 10.3390/v17040540 (PMC12031151; doi:10.3390/v17040540)
Supplement: Supplementary file 1 [file viruses-17-00540-s001.zip › viruses-3521768-supplementary.pdf]

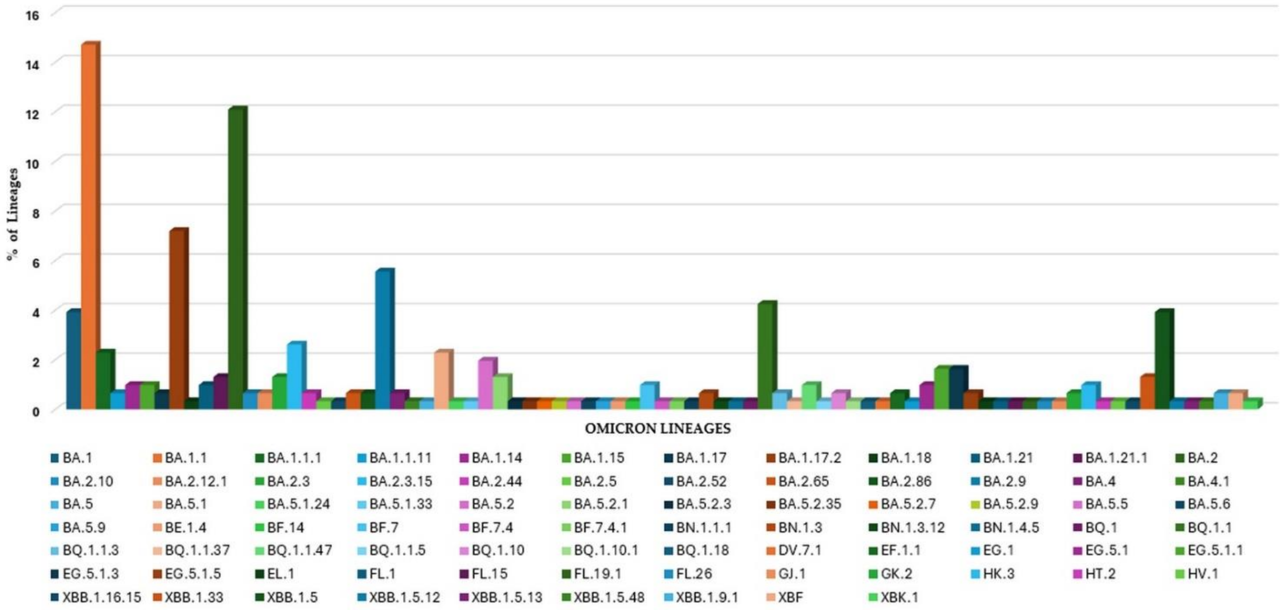

Figure S1. Distribution of Omicron lineages from December 2021 to December 2023. Distribution of variants according to Nextstrain Clades and Pangolin for 306 SARS-CoV-2 whole genome sequences collected from individuals attending the University Hospital of Rome Tor Vergata in Central Italy.

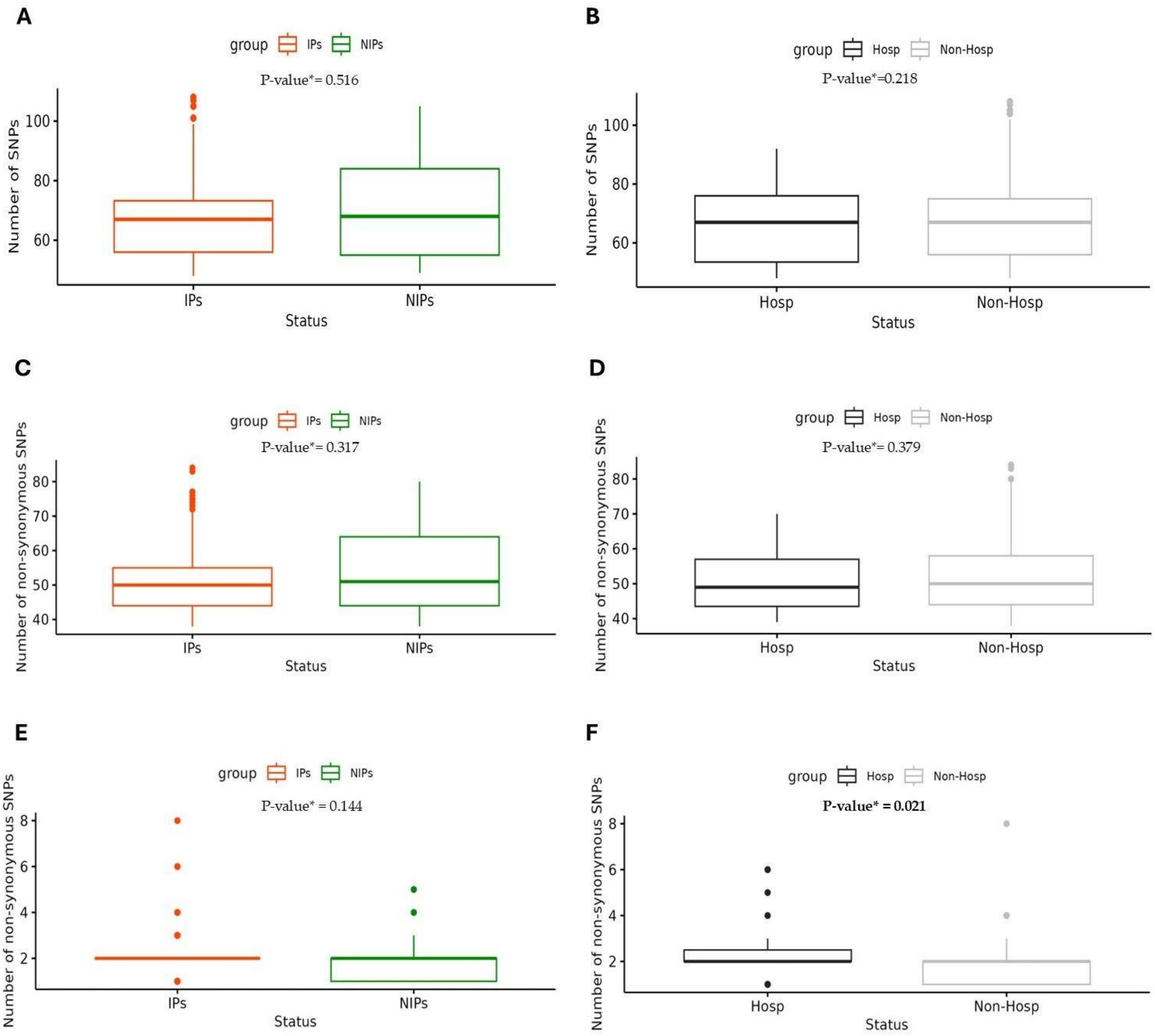

Figure S2. Median number and Interquartile range of high-abundant SNPs (frequency ≥ 40%) observed in IPs versus NIPs and in Hospitalized versus Non-Hospitalized individuals (**A**) and (**B**), respectively; non-synonymous abundant SNPs (frequency ≥ 20%) observed in IPs versus NIPs and in Hospitalized versus Non-Hospitalized individuals (**C**) and (**D**), respectively. Non-synonymous low-abundant SNPs (frequency from 5% to 19%) observed in IPs versus NIPs and in Hospitalized versus Non-Hospitalized individuals (**E**) and (**F**), respectively. SNPs: single nucleotide polymorphism. * *P*-values were calculated by Kruskal-Wallis test. Significant *p*-value was reported in bold.

Table S1. Distribution of main five Omicron lineage groups.

Group Lineages	Lineage	N	% Intra-Population [a]	% Intra-Group [b]	Group Lineages	Lineages	N	% Intra-Population [a]	% Intra-Group [b]
(a) BA.1 n = 104 (34.0%)	**BA.1**	12	3.9	11.5	(d) BQ+BE+EF n = 28 (9.2%)	**BE.1.4**	1	0.3	3.6
	**BA.1.1**	45	14.7	43.3		**BQ.1**	1	0.3	3.6
	**BA.1.1.1**	7	2.3	6.7		**BQ.1.1**	13	4.2	46.4
	**BA.1.1.11**	2	0.7	1.9		**BQ.1.1.3**	2	0.7	7.1
	**BA.1.14**	3	1.0	2.9		**BQ.1.1.37**	1	0.3	3.6
	**BA.1.15**	3	1.0	2.9		**BQ.1.1.47**	3	1.0	10.7
	**BA.1.17**	2	0.7	1.9		**BQ.1.1.5**	1	0.3	3.6
	**BA.1.17.2**	22	7.2	21.2		**BQ.1.10**	2	0.7	7.1
	**BA.1.18**	1	0.3	1.0		**BQ.1.10.1**	1	0.3	3.6
	**BA.1.21**	3	1.0	2.9		**BQ.1.18**	1	0.3	3.6
	**BA.1.21.1**	4	1.3	3.8		**EF.1.1**	2	0.7	7.1
(b) BA.2+BA.4 n = 79 (25.8%)	**BA.2**	37	12.1	46.8	(e) Recombinants n = 62 (20.2%)	**BA.2.86**	2	0.7	3.2
	**BA.2.10**	2	0.7	2.5		**BN.1.1.1**	1	0.3	1.6
	**BA.2.12.1**	2	0.7	2.5		**BN.1.3**	2	0.7	3.2
	**BA.2.3**	4	1.3	5.1		**BN.1.3.12**	1	0.3	1.6
	**BA.2.3.15**	8	2.6	10.1		**BN.1.4.5**	1	0.3	1.6
	**BA.2.44**	2	0.7	2.5		**DV.7.1**	1	0.3	1.6
	**BA.2.5**	1	0.3	1.3		**EG.1**	1	0.3	1.6
	**BA.2.52**	1	0.3	1.3		**EG.5.1**	3	1.0	4.8
	**BA.2.65**	2	0.7	2.5		**EG.5.1.1**	5	1.6	8.1
	**BA.2.9**	17	5.6	21.5		**EG.5.1.3**	5	1.6	8.1
	**BA.4**	2	0.7	2.5		**EG.5.1.5**	2	0.7	3.2
	**BA.4.1**	1	0.3	1.3		**EL.1**	1	0.3	1.6
(c)BA.5 + BF n = 33 (10.8%)	**BA.5**	1	0.3	3.0		**FL.1**	1	0.3	1.6
	**BA.5.1**	7	2.3	21.2		**FL.15**	1	0.3	1.6
	**BA.5.1.24**	1	0.3	3.0		**FL.19.1**	1	0.3	1.6
	**BA.5.1.33**	1	0.3	3.0		**FL.26**	1	0.3	1.6
	**BA.5.2**	6	2.0	18.2		**GJ.1**	1	0.3	1.6
	**BA.5.2.1**	4	1.3	12.1		**GK.2**	2	0.7	3.2

	**BA.5.2.3**	1	0.3	3.0	**HK.3**	3	1.0	4.8
	**BA.5.2.35**	1	0.3	3.0	**HT.2**	1	0.3	1.6
	**BA.5.2.7**	1	0.3	3.0	**HV.1**	1	0.3	1.6
	**BA.5.2.9**	1	0.3	3.0	**XBB.1.16.15**	1	0.3	1.6
	**BA.5.5**	1	0.3	3.0	**XBB.1.33**	4	1.3	6.5
	**BA.5.6**	1	0.3	3.0	**XBB.1.5**	12	3.9	19.4
	**BA.5.9**	1	0.3	3.0	**XBB.1.5.12**	1	0.3	1.6
	**BF.14**	1	0.3	3.0	**XBB.1.5.13**	1	0.3	1.6
	**BF.7**	3	1.0	9.1	**XBB.1.5.48**	1	0.3	1.6
	**BF.7.4**	1	0.3	3.0	**XBB.1.9.1**	2	0.7	3.2
	**BF.7.4.1**	1	0.3	3.0	**XBF**	2	0.7	3.2
					**XBK.1**	1	0.3	1.6

[a] % Intra-population: Percentage of each lineage calculated with respect to study population, n = 306. [b] % Intra-group: Percentage of each lineage calculated with respect to specific Omicron lineage group.

Table S2. Prevalence and reads frequency of high-abundant SNPs with significant different distribution between IPs vs. NIPs, and Hospitalized vs. Non-Hospitalized individuals.

Location	Mutation	SNP	Type of Mutation	Prevalence (N, %)									Reads Frequency (Median, IQR)				
				IPs	NIPs	*P*-Value *	BA.1 N = 104	BA.2 + BA.4 N = 79	BA.5 + BF N = 33	BQ + BE + EF N = 28	Recombinants N = 62	*P*-Value *	BA.1 N = 104	BA.2 + BA.4 N = 79	BA.5 + BF N = 33	BQ + BE + EF N = 28	Recombinants N = 62
Envelope	T11A	A26275G	Non-Syn	28 (14.9)	32 (27.1)	0.009	0	0	0	0	60 (96.8)	<0.0001	—	—	—	—	99.7 (97.7–99.9)
RdRp	G662S	G15451A	Non-Syn	28 (14.9)	32 (27.1)	0.009	0	0	1 (3.0)	0	59 (95.2)	<0.0001	—	—	99.9 (99.8–100)	—	99.9 (99.8–100)
Spike	G252V	G22317T	Non-Syn	12 (6.4)	17 (14.4)	0.020	0	0	0	0	29 (46.8)	<0.0001	—	—	—	—	100 (99.8–100)
Spike	G339D	G22578A	Non-Syn	157 (83.5)	86 (72.9)	0.025	104 (100)	78 (98.7)	33 (100)	28 (100)	0	<0.0001	99.9 (99.7–100)	99.9 (99.7–100)	99.9 (99.7–100)	99.9 (99.7–100)	—
Spike	G339H	G22577C	Non-Syn	29 (15.4)	31 (26.3)	0.020	0	0	0	0	60 (96.8)	<0.0001	—	—	—	—	100 (100–100)
Spike	I410I	C22792T	Syn	27 (14.4)	7 (5.9)	0.022	0	34 (43.0)	0	0	0	<0.0001	—	99.6 (99.1–99.7)	—	—	—
Spike	G446S	G22898A	Non-Syn	68 (36.2)	57 (48.3)	0.036	63 (60.6)	0	0	0	62 (100)	<0.0001	99.9 (99.8–100)	—	—	—	99.9 (99.8–100)
Spike	N460K	T22942G	Non-Syn	28 (14.9)	32 (27.1)	0.009	0	0	0	0	60 (96.8)	<0.0001	—	—	—	—	100 (99.9–100)
Spike	F490S	T23031C	Non-Syn	27 (14.4)	32 (27.1)	0.006	0	0	0	0	59 (95.2)	<0.0001	—	—	—	—	99.9 (99.8–100)

Location	Mutation	SNP	Type of Mutation	Prevalence (N, %)									Reads Frequency (Median, IQR)				
				Hosp	Non-Hosp	*P*-Value *	BA.1 N = 104	BA.2 + BA.4 N = 79	BA.5 + BF N = 33	BQ + BE + EF N = 28	Recombinants N = 62	*P*-Value *	BA.1 N = 104	BA.2 + BA.4 N = 79	BA.5 + BF N = 33	BQ + BE + EF N = 28	Recombinants N = 62
nsp3	G1001S	G5720A	Non-Syn	0 (0.0)	26 (10.0)	0.020	0	0	0	0	26 (41.9)	<0.0001	—	—	—	—	99.9 (99.9–100)
nsp14	I42V	A18163G	Non-Syn	37 (78.7)	242 (93.4)	0.001	93 (89.4)	75 (94.9)	31 (93.9)	26 (92.9)	54 (87.1)	0.480	100 (99.9–100)	100 (99.9–100)	100 (99.9–100)	100 (99.9–100)	100 (99.9–100)
nsp15	T112I	C19955T	Non-Syn	19 (40.4)	159 (61.4)	0.007	0	77 (97.5)	28 (84.9)	25 (89.3)	48 (77.4)	<0.0001	—	100 (100–100)	100 (100–100)	100 (100–100)	100 (100–100)
nsp15	E145E	A20055G	Syn	18 (38.3)	155 (59.8)	0.006	0	77 (97.5)	27 (81.8)	25 (89.3)	44 (71.0)	<0.0001	—	100 (99.9–100)	100 (99.9–100)	100 (99.9–100)	100 (99.9–100)
Nucleocapsid	N8N	T28297C	Syn	0 (0.0)	27 (10.4)	0.021	0	0	0	0	27 (43.6)	<0.0001	—	—	—	—	100 (99.8–100)
Spike	T376A	A22688G	Non-Syn	23 (48.9)	172 (66.4)	0.022	1 (1.0)	78 (98.7)	32 (97.0)	28 (100)	56 (90.3)	<0.0001	99.9 (99.7–100)	99.9 (99.7–100)	99.9 (99.7–100)	99.9 (99.7–100)	99.9 (99.7–100)

Abbreviations: SNP: Single Nucleotide Polymorphism; IPs: Immunocompromised People; NIPs: Non-Immunocompromised People; Hosp: Hospitalized individuals; nsp: non-structural protein; RdRp: RNA-dependent RNA polymerase; IQR: interquartile range. Prevalence and read frequency are expressed as N (%) and median (interquartile range), respectively. * *P*-values were calculated by Chi-square test, Fisher's exact test, or Chi-square test for trend, as appropriate.

Table S3. Prevalence and type of mutation of 109 SNPs involved in significant positive or negative associations in pairs.

SNP	Type of Mutation	Prevalence * n (%)	Mutation	Gene
**A26275G**	Non-Synonymous	60 (19.6)	T11A	E
**G26529A**	Non-Synonymous	64 (20.9)	D3N	M
**A26530G**	Non-Synonymous	103 (33.7)	D3G	M
**C26858T**	Synonymous	141 (46.1)	F112F	M
**G28681T**	Non-Synonymous	28 (9.2)	E136D	N
**A29510C**	Non-Synonymous	202 (66.0)	S413R	N
**C28312T**	Non-Synonymous	29 (9.5)	P13L	N
**A28330G**	Synonymous	21 (6.9)	G19G	N
**G28936T**	Non-Synonymous	113 (36.9)	L221F	N
**T28297C**	Synonymous	27 (8.8)	N8N	N
**A405G**	Non-Synonymous	51 (16.7)	K47R	nsp1
**T670G**	Non-Synonymous	201 (65.7)	S135R	nsp1
**T13195C**	Synonymous	104 (34.0)	V57V	nsp10
**G16935A**	Non-Synonymous	28 (9.2)	M233I	nsp13
**A17039G**	Non-Synonymous	24 (7.8)	N268S	nsp13
**C17410T**	Non-Synonymous	202 (66.0)	R392C	nsp13
**T16342C**	Non-Synonymous	52 (17.0)	S36P	nsp13
**T17859C**	Synonymous	50 (16.3)	Y541Y	nsp13
**A16878T**	Synonymous	20 (6.5)	T214T	nsp13
**A19326G**	Synonymous	51 (16.7)	P429P	nsp14
**A18492G**	Synonymous	21 (6.9)	P151P	nsp14
**A19743T**	Non-Synonymous	55 (18.0)	E41D	nsp15
**C19955T**	Non-Synonymous	178 (58.2)	T112I	nsp15
**A20055G**	Synonymous	173 (56.5)	E145E	nsp15
**G19741A**	Non-Synonymous	55 (18)	E41K	nsp15
**C1931A**	Non-Synonymous	28 (9.2)	Q376K	nsp2
**C2470T**	Synonymous	36 (11.8)	A555A	nsp2
**C2334T**	Non-Synonymous	19 (6.2)	A510V	nsp2
**C1627T**	Synonymous	23 (7.5)	L274L	nsp2
**C2790T**	Non-Synonymous	202 (66.0)	T24I	nsp3
**G4184A**	Non-Synonymous	202 (66.0)	G489S	nsp3
**C4321T**	Synonymous	202 (66.0)	A534A	nsp3
**A2832G**	Non-Synonymous	104 (34.0)	K38R	nsp3
**C3241T**	Synonymous	22 (7.2)	D174D	nsp3
**T5386G**	Synonymous	104 (34.0)	A889A	nsp3
**G8393A**	Non-Synonymous	107 (35.0)	A1892T	nsp3
**G5720A**	Non-Synonymous	26 (8.5)	G1001S	nsp3
**T2954C**	Synonymous	27 (8.8)	L79L	nsp3
**G5924A**	Non-Synonymous	24 (7.8)	V1069I	nsp3
**C9344T**	Non-Synonymous	200 (65.4)	L264F	nsp4
**A9424G**	Synonymous	200 (65.4)	V290V	nsp4
**C9534T**	Non-Synonymous	202 (66.0)	T327I	nsp4
**C9866T**	Non-Synonymous	133 (43.5)	L438F	nsp4
**C9693T**	Non-Synonymous	19 (6.2)	A380V	nsp4
**C10198T**	Synonymous	202 (66.0)	D48D	nsp5
**G10447A**	Synonymous	202 (66.0)	R131R	nsp5
**C11750T**	Non-Synonymous	30 (9.8)	L260F	nsp6
**A11537G**	Non-Synonymous	104 (34.0)	I189V	nsp6
**G12160A**	Synonymous	64 (20.9)	E23E	nsp8

**C12880T**	Synonymous	202 (66.0)	I65I	nsp9
**C12789T**	Non-Synonymous	29 (9.5)	T35I	nsp9
**C29625T**	Non-Synonymous	36 (11.8)	S23F	ORF10
**C26060T**	Non-Synonymous	202 (66.0)	T223I	ORF3a
**C25416T**	Synonymous	70 (22.9)	F8F	ORF3a
**A27259C**	Synonymous	245 (80.1)	R20R	ORF6
**G27382C**	Non-Synonymous	141 (46.1)	D61L	ORF6
**A27383T**	Non-Synonymous	141 (46.1)	D61L	ORF6
**T27384C**	Non-Synonymous	144 (47.1)	D61L	ORF6
**A27507C**	Synonymous	20 (6.5)	G38G	ORF7a
**G27915T**	Non-Synonymous	46 (15.0)	G8STOP	ORF8
**T14257C**	Non-Synonymous	27 (8.8)	Y264H	RdRp
**C15714T**	Synonymous	202 (66.0)	L749L	RdRp
**C15240T**	Synonymous	105 (34.3)	N591N	RdRp
**C15738T**	Synonymous	53 (17.3)	F757F	RdRp
**G15451A**	Non-Synonymous	61 (19.9)	G662S	RdRp
**T15939C**	Synonymous	51 (16.7)	D824D	RdRp
**C21618T**	Non-Synonymous	202 (66.0)	T19I	S
**G21987A**	Non-Synonymous	140 (45.8)	G142D	S
**G22599C**	Non-Synonymous	148 (48.4)	R346T	S
**C22686T**	Non-Synonymous	302 (98.7)	S375F	S
**A22688G**	Non-Synonymous	195 (63.7)	T376A	S
**G22775A**	Non-Synonymous	202 (66.0)	D405N	S
**A22786C**	Non-Synonymous	202 (66.0)	R408S	S
**G22813T**	Non-Synonymous	265 (86.6)	K417N	S
**A22893C**	Non-Synonymous	30 (9.8)	K444T	S
**T22917G**	Non-Synonymous	71 (23.2)	L452R	S
**T22942A**	Non-Synonymous	90 (29.4)	N460K	S
**A23013C**	Non-Synonymous	304 (99.3)	E484A	S
**G23642T**	Non-Synonymous	124 (40.5)	A694S	S
**C21846T**	Non-Synonymous	104 (34.0)	T95I	S
**A23040G**	Non-Synonymous	181 (59.2)	Q493R	S
**G23048A**	Non-Synonymous	105 (34.3)	G496S	S
**C23202A**	Non-Synonymous	107 (35)	T547K	S
**C23664T**	Non-Synonymous	23 (7.5)	A701V	S
**C24130A**	Non-Synonymous	104 (34.0)	N856K	S
**C24503T**	Non-Synonymous	104 (34.0)	L981F	S
**T22673C**	Non-Synonymous	103 (33.7)	S371L	S
**C22480T**	Synonymous	20 (6.5)	F306F	S
**T22942G**	Non-Synonymous	90 (29.4)	N460K	S
**G22577C**	Non-Synonymous	61 (19.9)	G339H	S
**T21810C**	Non-Synonymous	51 (16.7)	V83A	S
**C22000A**	Non-Synonymous	50 (16.3)	H146Q	S
**C22109G**	Non-Synonymous	51 (16.7)	Q183E	S
**T22200A**	Non-Synonymous	201 (65.7)	V213E	S
**G22317T**	Non-Synonymous	29 (9.5)	G252V	S
**C22664A**	Non-Synonymous	50 (16.3)	L368I	S
**G22895C**	Non-Synonymous	54 (17.6)	V445P	S
**T23018C**	Non-Synonymous	120 (39.2)	F486P	S
**T22896C**	Non-Synonymous	53 (17.3)	V445P	S
**T23019C**	Non-Synonymous	57 (18.6)	F486P	S
**G21718T**	Non-Synonymous	19 (6.2)	Q52H	S

**T22930A**	Non-Synonymous	19 (6.2)	F456L	S
**T23018G**	Non-Synonymous	120 (39.2)	F486V	S
**T22200G**	Non-Synonymous	201 (65.7)	V213G	S
**T23031C**	Non-Synonymous	61 (19.9)	F490S	S
**T22679C**	Non-Synonymous	302 (98.7)	S373P	S
**T22882G**	Non-Synonymous	265 (86.6)	N440K	S
**C23604A**	Non-Synonymous	306 (100)	P681H	S
**G22599A**	Non-Synonymous	148 (48.4)	R346K	S

Abbreviations: SNP: Single Nucleotide Polymorphism; E: Envelope; M: Membrane; N: Nucleocapsid; nsp: non-structural protein; ORF: open reading frame; RdRp: RNA-dependent RNA polymerase; S: Spike. * Prevalence calculated on 306 individuals.

Table S4. Prevalence of 19 SNPs involved in three clusters across the five main Omicron lineage groups.

Cluster	SNP	Amino Acid Residue	Type of Mutation	Omicron Lineage Groups Prevalence n (%)				
				BA.1	BA.2 + BA.4	BA.5 + BF	BQ + BE+ EF	Recombinants
**Cluster 1**	G23642T	S: A694S	Non-Synonymous	24 (23.1)	45 (57.0)	16 (48.5)	13 (46.4)	33 (53.2)
	T15474G	RdRp: G678G	Synonymous	32 (30.8)	47 (59.5)	15 (45.5)	13 (46.4)	0 (0.0)
	G28936T	N: L221F	Non-Synonymous	43 (41.3)	47 (59.5)	17 (51.5)	14 (50.0)	24 (38.7)
**Cluster 2**	A2832G	nsp3: K38R	Non-Synonymous	104 (100)	0 (0.0)	0 (0.0)	0 (0.0)	0 (0.0)
	T5386G	nsp3: A889A	Synonymous	104 (100)	0 (0.0)	0 (0.0)	0 (0.0)	0 (0.0)
	G8393A	nsp3: A1892T	Non-Synonymous	104 (100)	0 (0.0)	1 (0.96)	0 (0.0)	2 (1.9)
	A11537G	nsp6: I189V	Non-Synonymous	104 (100)	0 (0.0)	0 (0.0)	0 (0.0)	0 (0.0)
	T13195C	nsp10: V57V	Synonymous	104 (100)	0 (0.0)	0 (0.0)	0 (0.0)	0 (0.0)
	C15240T	RdRp: H599H	Synonymous	104 (100)	0 (0.0)	1 (0.96)	0 (0.0)	0 (0.0)
	C21846T	S: T95I	Non-Synonymous	104 (100)	0 (0.0)	0 (0.0)	0 (0.0)	0 (0.0)
	G23048A	S: G496S	Non-Synonymous	104 (100)	0 (0.0)	0 (0.0)	0 (0.0)	0 (0.0)
	C23202A	S: T547K	Non-Synonymous	104 (100)	1 (0.96)	2 (1.9)	0 (0.0)	0 (0.0)
	C24130A	S: N856K	Non-Synonymous	104 (100)	0 (0.0)	0 (0.0)	0 (0.0)	0 (0.0)
	C24503T	S: L981F	Non-Synonymous	104 (100)	0 (0.0)	0 (0.0)	0 (0.0)	0 (0.0)
	A26530G	M: D3G	Non-Synonymous	102 (98.1)	0 (0.0)	0 (0.0)	0 (0.0)	1 (0.96)
	T22673C	S: S371L	Non-Synonymous	103 (99.0)	0 (0.0)	0 (0.0)	0 (0.0)	0 (0.0)
**Cluster 3**	G28936T	N: L221F	Non-Synonymous	24 (23.1)	45 (57.0)	16 (48.5)	13 (46.4)	33 (53.2)
	G23642T	S: A694S	Non-Synonymous	63 (41.3)	47 (59.5)	17 (51.5)	14 (50.0)	24 (38.7)
	T23620G	S: S686R	Non-Synonymous	43 (60.6)	56 (70.9)	17 (51.5)	19 (38.7)	38 (61.3)

Abbreviations: SNP: Single Nucleotide Polymorphism.
